# Improving paediatric quality of care through a Sweden–Uganda twinning partnership – reflections on implementation and early experiences from the child thrive project

**DOI:** 10.1080/16549716.2026.2729905

**Published:** 2026-10-01

**Authors:** Helena Hildenwall, Dorothy Akongo, Emmanuel Tenywa, Svante Norgren, Moses Kyangwa, Alfred Yayi, William Mugowa Waibi, Viveka Nordberg, Agnes Linnér, Eva Svensson, Harriet Nambuya, Erik Larsson, Aziz Kiwanuka, Charlotte Elf, Brenda Ocokoru, Maburuka Anguparu, Deogratias Migadde, Peter Waiswa

**Affiliations:** a Pediatric Health Initiative, Stockholm, Sweden; b Astrid Lindgren Children’s Hospital, Karolinska University Hospital, Stockholm, Sweden; c Department of Global Public Health, Karolinska Institutet, Stockholm, Sweden; d Busoga Health Forum, Jinja, Uganda; e Department of Paediatrics and Child Health, Jinja Regional Referral Hospital, Jinja, Uganda; f Jinja Regional Referral Hospital, Jinja, Uganda; g Reproductive and Child Health Department, Ministry of Health, Kampala, Uganda; h Department of Health Policy, Planning and Management, Makerere University School of Public Health, Kampala, Uganda

**Keywords:** Low-income countries, international collaboration, quality of care, twinning partnership, capacity building, health system strenghtening

## Abstract

Despite major global progress in child health, significant inequalities remain between low- and high-income countries. In Uganda’s Busoga region, child mortality is still high, largely due to preventable conditions and limited healthcare resources. In response, a paediatric health partnership was established in 2021 between the Busoga region and Stockholm, Sweden. Following a quality-of-care assessment in two Ugandan hospitals, the collaboration identified four priority areas: paediatric emergency care, neonatology, child neurology with a focus on epilepsy, and child rights. Over 3 years, 63 staff exchanges have taken place, enabling joint reviews of clinical practices and identification of improvement needs. A key achievement is the implementation of the WHO Emergency Triage, Assessment and Treatment Plus (ETAT+), supported by training 14 national facilitators who now serve as focal persons in their facilities. Initiatives in neonatology have strengthened early interventions for premature and low birth weight infants such as improved nursing care, infection prevention, and nutrition practices. In neurology, the introduction of an epilepsy registry and education programmes has reduced stigma and improved management. The child rights initiative has trained more than 80 ambassadors and fostered child-friendly hospital environments that promote participation. The in-hospital mortality dropped over the project period (2020–2025) from 26.4% to 21.7% for neonates (*p* = 0.021) and from 3.8% to 2.8% (*p* =  <0.01) for children less than five outside the neonatal period. Despite resource constraints, the partnership has benefited both regions. This twinning model highlights how equitable international collaborations can sustainably advance child health and reinforce healthcare systems in diverse settings.

## Paper Context

- **Main findings**: We describe a Sweden–Uganda hospital twinning partnership that improved paediatric quality of care across four clinical areas, with in-hospital child mortality falling over the project time: from 26.4% to 21.7% for neonates (p = 0.021) and from 3.8% to 2.8% (p =  <0.01) for children less than five outside the neonatal period.
- **Added knowledge**: A structured twinning model combining baseline assessment, implementation science, and a train-the-trainer approach can systematically close the know-do gap in paediatric care in resource-limited settings.
- **Global health impact for policy and action**: Sustainable international health partnerships require long-term institutional commitment, explicit local ownership, and a deliberate division of labour where high-income country partners support evidence synthesis while low-income country partners lead contextualised implementation.

## Background

Unacceptable disparities in global progress in child health remain between low- and high-income countries. The quality of healthcare in low-income countries is often limited by high patient volumes, lack of access to diagnostic equipment, and shortages of trained personnel. Insufficient quality of care has been recognized to cause 60% of premature mortality in low-income countries and leads to preventable morbidity, limiting participation in education, work, and society [1]. Children are affected by well-described sub-standard neonatal and emergency care, which can contribute to mortality and, for survivors, long-term neurodevelopmental impairments that affect their ability to succeed in school [2].

A steady decline in infant and under-five mortality has been observed in Uganda over the years. Still, the under-five mortality rate for Uganda remains high at 50 deaths per 1000 live births in 2022 [3]. The slow reduction has been attributed to institutional challenges in the health sector, including inadequate technical capacity. This is largely due to insufficient continuous medical education, poor adherence to guidelines, and a critical shortage of staff, equipment, and supplies. Maintaining adherence to established protocols and upholding the quality of care remains a significant challenge. Studies indicate that less than 33% of sick children are checked for established danger signs, with factors such as time since training, the presence of higher-qualified staff, and the child’s age influencing adherence to protocols [4]. Furthermore, shortages of drugs, inadequate training, lack of essential equipment and knowledge, coupled with high patient loads, patient delays, frequent drug stock-outs, and the inability to provide 24-h staffing, all contribute to the poor quality of care [5,6]. Overall post-discharge mortality remains high during the first 6 months following hospitalisation, highlighting the need for improved discharge counselling and follow-ups [7–9]. Despite good evidence for interventions that can reduce child mortality and morbidity, the lack of implementation support and staff limitations impair the closure of the know-do gap in paediatric care. To address the gap between evidence-based practices and care delivery, a partnership was established to enhance paediatric care quality by connecting paediatric health care workers from low- and high-income settings through collaborative improvement efforts.

## Methods

This paper is reported as a descriptive case study of an institutional twinning partnership that encompassed multiple, concurrently evolving activities across training, mentorship, and health-system strengthening, without a single pre-specified intervention, process measure, or implementation strategy. Data presented were collected prospectively for programme monitoring and donor reporting and are synthesised narratively to describe the partnership’s structure, activities, and outcomes over time.

### Settings

This project includes two hospitals as the main collaborators: Jinja Regional Referral Hospital (JRRH) in Uganda and Astrid Lindgren Children’s Hospital (ALB) at Karolinska University Hospital in Sweden. JRRH is the main public hospital in the Busoga region of eastern Uganda and one of the biggest public hospitals in Uganda, with 6–7000 babies born and 7–9000 admissions of children less than 5 years of age every year. It serves as a regional referral centre and consequently receives patients from the entire region. The hospital also acts as the main teaching hospital for the lower-level health facilities in the Busoga region and hosts large numbers of students (medical students, postgraduate students, nurse students) from within and outside the region.

Astrid Lindgren Children’s Hospital is the paediatric centre within Karolinska University Hospital in Stockholm, Sweden, providing specialized and highly specialized care for children from newborns to adolescents, both nationally and internationally. The hospital is strongly connected to medical research and education through Karolinska Institutet and handles a large volume of outpatient and inpatient visits each year.

## Initial work

In autumn 2021, a paediatric healthcare partnership was established, driven by the nonprofit organizations Pediatric Health Initiative, Sweden (www.pediatrichealthinitiative.org) and Busoga Health Forum, Uganda (www.busogahealthforum.org). Through funding from the International Centre for Local Democracy in Sweden (www.icld.se), the collaboration quickly developed into a regional partnership between Jinja City in Uganda and Region Stockholm, with the region’s two main hospitals Jinja Regional Referral Hospital in Uganda and Astrid Lindgren Children’s Hospital in Sweden, as central partners. During the inception year, activities were conducted to define the partnership goals and focus areas. Steering groups were established in both countries, including representatives from the included NGOs, hospital directors, and regional politicians from both majority and opposition parties, to ensure long-term support and sustainable implementation. In line with the funder’s core values and in agreement with all partners involved, the aspects of equity, participation, transparency, and accountability were considered and integrated into all projects. Data collection was performed to map the current quality of care using the World Health Organization’s ‘Quality of Hospital Care’ assessment tools for children, newborns, and maternal health [10,11]. The tools involve staff interviews, observations, and file reviews to assess the quality of care across hospital support services, patient case management, policies, and organisation of services. A multidisciplinary Swedish–Ugandan team, including paediatricians, clinical officers, nurses, and social scientists, collected data from Jinja Regional Referral Hospital on different components of the WHO tool according to their area of expertise. Findings were subsequently discussed jointly by the full team, and scores were assigned by consensus, consistent with the participatory, team-based approach intended by the WHO tool. The scores are defined as follows: *3 = care corresponding to international standards*, 2 = *substandard care but no significant hazard to health or violation of human rights*, 1 = *inadequate care with consequent serious health hazards or violation of children’s rights,* and 0 = *very poor care with consequent systematic and severe hazards to the health of children*. The assessment of maternal and newborn care used a percentage for scoring of standards met (0–100%) that was subsequently translated into an overall score between 0–3 as per the above classifications: 0 = <50% of standards met, 1 = 50–65%, 2 = 66–79%, 3 = ≥80%.

## Results of the initial assessment

The paediatric in-hospital case fatality rate for children less than five outside the neonatal was 3.8% among 6553 admissions in 2020. For the same year, mortality among 689 neonatal admissions was 26.4%. The main mortality causes as registered in hospital data were pneumonia, malaria, sickle cell disease and septicaemia, while for neonatal admissions, it was prematurity, neonatal sepsis and ‘other neonatal complications’.

Tables 1 and 2 provide an overview of the assessment results for areas of paediatric and newborn hospital care. For paediatric care, no area reached a score of three, which corresponds to care according to international standards. Most areas scored around 2, with children’s rights and ward infrastructure achieving the lowest scores of 1.7 and 1.4, respectively. For newborn care, the lowest scores were seen for advanced newborn care (21%), pharmacy management (44%) and ward infrastructure (43%), which all scored below the required 50% and were thus classified as ‘very poor care’.

**Table 1.** Scoring quality for areas of paediatric care.

Area	Theme	Subchapter score (0–3)	Strengths	Weaknesses
Overall infrastructure	Physical structure	2.3	Backup systems for power and water	Staff limitations, overcrowding
	Statistics	2.0	Biostatisticians available	Data not actively used for quality improvement, limited information in medical records, unclear treatment charts
	Pharmacy management	1.8	Essential drugs list available	Frequent drug stock outs, challenges to maintain cold chain
	Equipment and supplies	2.0	Most items available at assessment	Equipment not well maintained and organised
	Diagnostic services	2.3	Basic tests available	Lack of glucometers, limited use of blood cultures
	Ward infrastructure	1.4	Child friendly posters available	Overcrowded, Intensive Care Unit at distance, no playroom available
Case management	Emergency management	2.6	Triage system in place, oxygen available	Untimely assessments, especially during night, staff not trained in ETAT, intermittent lack of drugs, issues too differentiate types of shock
	Case management of respiratory diseases	2.4	Oxygen and nebulisers available, pulse oximeters available	Preference for ceftriaxone to all, oxygen monitoring not done
	Case management of diarrhoea	2.7	Dehydration plans well known	Overuse of antibiotics
	Case management of other conditions presenting with fever	2.0	Early suspicion of TB	Lumbar puncture rarely done, no diagnostics available for urinary tract infections, overuse of antibiotics
	Case management of anaemia and growth failure	2.2	Availability of nutrition unit and nutritionists	Nutritional assessments not available in-patient files
	Case management of chronic conditions	2.0	HIV routines in place	Developmental assessments not done, no registers for chronic patients
	Supportive care	2.5	Complementary feeding free of charge (under project)	Deficits in monitoring of intravenous fluids, pain management and oxygen. No toys/activities available for admitted children
	Monitoring and follow up	1.8	Monitoring charts available	Monitoring charts not used, limited reassessments
	Infection prevention	1.8	Isolation rooms available	Soap, sanitizers and gloves often missing, patient crowding
Policies and organisation of services	Guidelines, training and audits	1.9	Guidelines available at lab,	Job aids not available, mortality audits not done regularly, no regular in-service training
	Access to hospital	2.3	Hospital services open to all and free of charge	Need for guardians to pay for medicines/equipment when missing
	Children’s right to respectful and holistic care	1.7	Hospital services open to all	Child rights not visible in facility, procedures not done in separate spaces, no toys/playroom, pain not monitored/treated

**Table 2.** Scoring quality for areas of newborn care, score scale: 0 = <50% of the standards met, 1 = 50–65%, 2 = 66–79%, 3 = ≥80%.

Area	Theme	Score 0–3(%)	Strengths	Weaknesses
Overall infrastructure	HR policies	3 (84%)	National staffing standards implemented.	No programme for continuing professional development.
	Pharmacy management/medicine availability	0 (44%)	Intravenous fluids available.	Key medicines frequently out of stock Storage conditions inadequate.
	Equipment availability	1 (61%)	Incubators available. Oxygen source available. Blood-sugar testing sticks, infusion sets, and IV cannulas available.	No CPAP. Some equipment non-functional. Blankets, cord ties, and spinal needles unavailable. Masks and prongs reused. No NGT for preterm.
	Equipment functionality and knowledge	1 (60%)	Pulse oximetry used for all preterm infants on oxygen.	Some equipment present but not functioning or not used correctly. Knowledge gaps in correct use of equipment.
	Laboratory support	2 (76%)	Quality control system in place.	Blood-gas analysis, CRP, urine-protein, and coagulation tests unavailable. Blood not available at all times.
	Ward infrastructure	0 (43%)	Dedicated KMC area. Service areas in proximity to labour rooms. Handwashing facilities available.	Emergency areas not well designed. No adequate privacy. Insufficient toilets and food preparation areas.
Hospital policies and organisation of services	Infection prevention	2 (76%)	Awareness posters on walls. Reliable water source. Appropriate waste management. Written handwashing procedures posted.	No up-to-date infection control guidelines. Staff not trained yearly on transmission precautions. Nosocomial transmission precautions undermined by overcrowding.
	Organisation of services for quality improvement	0 (46%)	Written plan for improving quality of care exists.	Guidelines not easily accessible at point of care. No reference material on ward. Audits and case reviews rarely performed.
	Rights of newborn	1 (57%)	Emergency care available 24/7. No discrimination by race, ethnicity, culture, or religion. Consent forms present. Patients treated with respect.	Basic equipment and supplies not always available. Care not consistently at international standard. Health education materials not always available. Privacy not ensured.
Case management newborn	Newborn care soon after birth	1 (58%)	Protocols for essential newborn care and resuscitation available. Immediate drying, skin-to-skin and breastfeeding encouraged.	No pre-warmed area. Blankets and hats not provided. No quiet/private atmosphere. Resuscitation equipment not always ready. Not always doctor review before discharge.
	Care of the healthy newborn	2 (75%)	Mother-infant separation minimised. No infant formula advertising. Exclusive breastfeeding recommended. Malformations checked.	Vital signs not recorded on day 1. Urine/stool output not monitored. Jaundice prevention inadequate. Discharge counselling brief.
	Care of premature and LBW infant	1 (52%)	Dedicated KMC area for clinically stable patients. Pulse oximetry used for all preterm infants on oxygen.	No up-to-date protocols for small preterm infants. Lack of trained staff. KMC times and observations not recorded. No hats or socks. Suboptimal nutrition – IV fluid only, No milk bank, no syringe drivers, no parenteral nutrition. No growth charts. Cannot manage RDS (no surfactant, no CPAP).
	Care of the sick newborn	1 (57%)	First-line antibiotics available. Mothers supported to maintain milk supply. No routine separation. Infants assessed for danger signs. Oxygen provided when indicated.	Monitoring inadequate. No up-to-date sepsis protocols. Blood cultures rarely taken. No pain or anxiety management. No parenteral nutrition. Severe respiratory failure cannot be treated. Electrolytes cannot be checked. Antibiotic dosing not adjusted for gestational age. No growth charts.
	Advanced newborn care	0 (21%)	Blood transfusions can be administered.	Insufficient personnel with relevant training. No regular skills training, diagnostics, or monitoring systems. No parenteral nutrition or central catheters. Blood cultures seldom taken. No lumbar puncture needles. No growth charts. Environmental stress not minimised. No transport protocols for critically ill infants. Referral and follow-up pathway for high-risk infants unclear.

CPAP = Continuous Positive Airway Pressure, KMC = Kangaroo Mother Care, NGT = Naso-Gastric Tube, RDS = Respiratory Distress Syndrome.

## Activities conducted within the partnership

Based on the findings from the quality-of-care assessment and in consultation with the partnership steering group, the JRRH hospital director and on-site paediatricians, an action plan was developed with four project areas: paediatric emergency care, neonatology, child rights, and child neurology, with a focus on epilepsy. The full partnership received 3 years of funding from ICLD’s municipal partnership programme.

The project model is based on the principle of ‘learning by doing,’ where collaboration, joint development, and knowledge sharing are central. All four projects include not only medical learning but focus on the structured implementation of target behaviours for quality improvement and address democratic and rights-based dimensions to enhance patient involvement and understanding of care provided to them and consider the needs of the most vulnerable groups in society. A central component of this approach is a cascading train-the-trainer model, in which selected participants are supported to become local trainers within their own workplaces.

Implementation scientists have been involved to provide all project participants with a training workshop on the use of implementation strategies to deepen the understanding of requirements for successful behaviour change. The COM‑B model is a behaviour change framework that proposes that any behaviour (B) results from an interaction between Capability, Opportunity, and Motivation. In this model, Capability refers to an individual’s capacity to engage in the behaviour (e.g. knowledge and skills), Opportunity covers external factors that enable or prompt the behaviour (e.g. resources, social norms, environmental constraints), and Motivation includes both reflective processes (plans, intentions, beliefs) and automatic processes (habits, emotions) that direct behaviour. Together, COM‑B is typically used to identify which of these components need to be targeted when designing or adapting interventions. All project groups applied the COM-B model to understand requirements to achieve the desired behaviour as defined by their project objective. Research projects have also been linked to the individual projects to deepen understanding of issues and/or monitor progress [12–14]. Table 3 provides a summary of cross-cutting lessons.

**Table 3.** Summary of cross-cutting lessons from all partnership projects.

Cross-cutting lesson	Description
Cascading train-the-trainer model	All projects selected local participants as trainers/ambassadors, building sustainable local capacity beyond the project period.
Behaviour change framework (COM-B)	Each project applied COM-B to identify capability, opportunity, and motivation gaps, grounding interventions in implementation science.
Mutual learning, not aid	The partnership deliberately moved away from donor–recipient dynamics; both Swedish and Ugandan teams gained learnings
Rights-based and equity-centred approach	All projects integrated democratic values (equity, participation, transparency, accountability) as required by the funder.
Addressing the know-do gap	A shared challenge across all areas: having guidelines does not ensure adherence. All projects targeted structured implementation support.
Staff motivation as an enabler	Participants across projects reported increased professional satisfaction and sense of purpose, underlining motivation as a key driver of quality improvement.

### Improved paediatric emergency care through training and local capacity building

Despite advances in child health, paediatric emergency care in sub-Saharan Africa continues to face major challenges. Limited availability of trained personnel, essential medicines, and equipment often delays the timely and effective management of critically ill children [15]. Overburdened facilities, weak referral systems, and inadequate triage further compromise outcomes, especially in rural and resource-constrained settings. These systemic gaps highlight the need for practical, scalable approaches to strengthen emergency care delivery and reduce preventable child deaths.

In light of this and given that the Uganda Ministry of Health and the Uganda Paediatric Association were simultaneously developing national Ugandan ETAT+ guidelines with the aim of rolling out ETAT+ training across all health facilities in Uganda, the partnership identified support for the implementation of Emergency Triage, Assessment and Treatment Plus (ETAT+) in the Busoga Region as a central component of the initiative.

ETAT is a triage system originally developed by the World Health Organization for paediatric emergency care, specifically designed for low- and middle-income countries [16]. It was later expanded into ETAT+ by national paediatric associations and further adapted into an online training programme [17]. ETAT+ aims to rapidly identify children with life-threatening conditions, ensure appropriate prioritization, and provide prompt stabilizing treatment as soon as possible after arrival at the hospital.

Before the project began, an assessment of ETAT+ use and readiness was conducted across 14 public and private-not-for-profit healthcare facilities in the Busoga region. The results showed significant potential for improvement, particularly in targeted training, improved routines, and stronger teamwork [13]. In response, and in consultation with Uganda’s Ministry of Health, a structure for recurring training programs was established. ETAT+ training courses were conducted with Swedish paediatric emergency staff as lead facilitators with a key component being the nomination of Ugandan participants for ETAT+ instructor training to build long-term local capacity.

Nominees were participants who demonstrated strong interest and engagement and held central positions in paediatric emergency care at their workplaces at JRRH and across hospitals in the Busoga region. They were invited to spend 2 weeks in Sweden, participating in formal ETAT+ instructor training, clinical observation in paediatric emergency care, and seminars and workshops focused on patient monitoring, patient safety management and implementation strategies. After training, instructors had to successfully join an ETAT+ course as an instructor under training and once approved, they were assigned as ETAT+ focal persons in their facilities and included in the Ministry of Health’s national pool of trainers. Course materials were co-developed with the Ministry to ensure local adaptation and sustainability.

Implementation began at JRRH, where eight instructors have been included as national ETAT+ instructors. The JRRH instructor team has established a routine to train all new staff in ETAT+ when they start their rotations at the paediatric unit. To reduce on numbers referred to JRRH for emergency stabilisation, the work has since expanded to four district public hospitals, with six additional health workers included as national instructors and an additional nine awaiting final approval. The goal is to ensure local training capacity and strengthen adherence to ETAT+ guidelines at the regional and district level.

### Neonatology

Each year, over two million newborns die within their first month of life, and complications related to premature birth or low birth weight are the leading causes of death among children under 5 years of age, followed by deaths related to birth asphyxia. In Uganda, neonatal mortality is 22 per 1,000 live births in 2022 [18], still far from the sustainable development goal of less than 12 deaths per 1,000 live births that is to be reached by 2030. Neonatal deaths primarily occur among preterm and/or low birth weight infants, with hospital acquired infections, suboptimal nutrition and respiratory support contributing substantially to mortality.

Our baseline assessments of neonatal care quality in Jinja identified the primary challenges in infection control, adequate nutrition, thermal care and respiratory support. Based on these findings, topics highlighted within the partnership were the initial care following birth – the ‘golden hour,’ neonatal resuscitation, non-separation between the mother and the infant including skin-to-skin contact, bridging obstetric and neonatal care, safe vascular access handling, antibiotic stewardship, enteral nutrition including early trophic feeding, hypoglycaemia management, fluid balance and infant- and family-centred neurodevelopmental care. Infection prevention and control has been a key topic, with nested research on neonatal ESBL prevalence.

The neonatal project between Jinja and Stockholm emphasizes continuous peer-to-peer learning, encouraging collaboration both within and across the participating teams. Activities span online and on-site courses, clinical visits, bedside teaching, simulation training, and skills training integrated into daily care, addressing teamwork, family involvement, and change management alongside clinical topics. Two courses on essential and emergency neonatal care have been held at JRRH, training nearly 100 members of staff in the Busoga region. A neonatal fellowship program in Kampala, developed outside the partnership, involves partners who also train in Jinja to strengthen national capacity. Four visits to Jinja from Stockholm, and three visits to Stockholm from Jinja have been conducted, involving ten members of neonatal staff, each from the Ugandan and Swedish teams. To support ongoing exchange, the teams stay connected through online platforms, enabling regular cross-country communication and networking.

### Child neurology with focus on epilepsy

Up to 70% of people with epilepsy can achieve seizure control through accurate diagnosis and the use of cost-effective and widely available anti-epileptic drugs. Despite this, over 75% of people with epilepsy in low-income countries lack treatment, a phenomenon known as the ‘treatment gap’ [19]. In rural Uganda, the treatment gap is nearly 80%, with children under five most affected [20].

Participants in the neurology project established a patient registry for all children with epilepsy at the Jinja neurology clinic. This organized monitoring is crucial for tracking disease progression and adjusting medication as needed. To reduce stigma, regular information meetings are held for caregivers, and efforts are also directed toward schools – targeting teachers, children, and parents. The Ugandan team has also participated in radio programs to raise public awareness about epilepsy.

The work has strengthened the integration of care efforts, for example, increased collaboration between the nutrition unit and the neurology team, as many children with neurological disorders are at risk of malnutrition. Physiotherapists and occupational therapists have received support, and several webinars, trainings, and seminars in paediatric neurology have been organized. Paediatric neurologists from Sweden also provide specialist consultations as collegial support to their Ugandan counterparts.

Improved care quality and increased access to medical treatment and medication have strengthened patients’ ability to participate actively in society. Better information for caregivers about conditions and treatment options has increased motivation to seek care early. Public education initiatives targeting school staff and communities have improved knowledge about neurological conditions such as epilepsy, fostering understanding and inclusion.

### Child rights in healthcare

Children’s rights in paediatric healthcare face persistent challenges in low-income countries, including limited resources, weak protection systems, and barriers to children’s participation and access to justice [21]. In Uganda, despite ratifying the UN Convention on the Rights of the Child (CRC) [22] and embedding many child rights in national law, practical gaps remain in ensuring protection, participation, and equitable care, especially for the most marginalized children. Both Uganda and Sweden have ratified the CRC, demonstrating formal commitment, but implementation barriers such as underfunded welfare authorities and insufficient child-sensitive approaches continue to affect children’s rights in practice.

Strengthening children’s rights in healthcare and society is a central theme of the partnership. The child rights project aims to raise awareness of children’s rights among healthcare staff, caregivers, and civil society. Within the project, child rights ambassadors are trained, a role already established at Astrid Lindgren Children’s Hospital, to advocate for children’s rights within healthcare institutions. The project also includes creating play areas and activities for hospitalized children to promote well-being and a sense of safety.

So far, over 80 child rights ambassadors from JRRH, nearby health facilities, and local businesses have been trained. The training combines digital and in-person sessions. Over about 6 months, participants collect children’s voices and perspectives as part of their ambassador certification. The project has produced a structured training programme as a model for other regions and municipalities to strengthen and secure children’s rights in practice [14].

The hospital’s premises have also been improved: corridors and the main entrance have been repainted to create a more welcoming environment, and the play area has been renovated with new materials. Two Child Rights Awareness Weeks have been held, involving children, caregivers, local communities, healthcare providers, and hospital leadership. During the first week, Uganda’s first hospital-based Children’s Parliament was held, where children shared their views on the kind of healthcare environment that they desire.

### Partnership impact in Sweden

The partnership benefits not only the Busoga region and its children but also strengthens Swedish healthcare in several ways. Paediatric staff from Astrid Lindgren Children’s Hospital gain valuable experience of conditions that are rare in high-income health care, broadening their clinical competence and diagnostic skills. At the same time, Swedish staff learn from the clinical insight and resource efficiency of their Ugandan colleagues, an inspiration for innovation in Sweden and an experience directly relevant to Swedish emergency preparedness. Operating effectively under high patient loads and constrained resources is precisely the skill set required when health emergencies, a global threat, place unexpected strain on the Swedish system. Moreover, observing the potential consequences of suboptimal nutrition, respiratory support, triaging, adherence to guidelines, etc. deepens the understanding of the importance of these aspects also in the Swedish settings. Many participants report increased professional meaning and motivation, enriching individual careers while fostering a culture of reflection and knowledge exchange.

### Change in case fatality rates over the project period

The paediatric in-hospital case fatality rate for children less than five outside the neonatal period for 2025 was 2.8% among 9807 admissions [18], compared to 3.8% fatality rate for the same age group among 6553 admissions in 2020 (χ^2^ = 12.54, *p* < 0.001). For the neonatal admissions, the mortality was 26.4% among 689 admissions in 2020 compared to 21.7% among 1192 admissions in 2025 (χ^2^ = 5.34, *p* = 0.021). While we cannot establish a causal relationship between project activities and this reduction in hospital mortality, we acknowledge that this reduction took place during intense collaborative work to improve the quality of care and ensure timely evidence-based management of all sick children.

## Discussion

International health partnerships between high- and low-income countries, formalised by the WHO as Twinning Partnerships for Improvement (TPI) [23] –have a track record of meaningful progress [24]. The Busoga–Stockholm partnership is one example: in-hospital mortality at JRRH fell significantly during the project, despite rising admissions, though causal association cannot be established. The changed numbers may reflect a genuine quality improvement but could also stem from case-mix shifts tied to JRRH’s growing referral role, better case recording, or more admissions due to increased caregiver trust as the quality improved.

Strong institutional ties between Karolinska University Hospital, Jinja Regional Referral Hospital, and local health facilities provided the foundation, with long-term commitment integrating quality improvement work with research and practice. Central to the partnership’s effectiveness was a deliberate departure from traditional donor-recipient dynamics toward genuine collaboration, and local capacity building with a complementary division of labour: Swedish professionals, with greater time availability, synthesised emerging guidelines and research, while Ugandan counterparts – despite heavy patient loads – contributed the contextual expertise without which implementation would have remained superficial, reflecting the different structural constraints of HIC and LIC health systems.

Capacity built through this partnership has shown signs of sustainability and transfer, beyond the original programme. This was underpinned by close collaboration with Ugandan leads throughout, and by use of the COM-B model to understand local barriers, and requirements for desired practices to take hold – informing decisions on what to train, how to structure supervision, and other elements to enhance institutional ownership from the outset, rather than being added afterward. ETAT+ facilitators trained through this collaboration have been incorporated into Uganda’s Ministry of Health national trainer cadre and have gone on to deliver donor-funded training independently of this partnership. Transferability is illustrated by several examples: the Child Rights Ambassadors initiative has become a published toolbox for use by other local governments and health facilities [14]; Ugandan partners have trained colleagues in a region of Kenya to establish an equivalent programme; and a patient registry for epilepsy established during the neurology component continues to support follow-up and drug-compliance monitoring. These examples suggest that sustainability has depended less on continued external funding than on institutionalisation within government structures and local ownership of specific tools and models, achieved through sustained co-design with local leads rather than externally imposed design.

The policy implications follow: Effective international health partnerships must address the ‘time poverty’ of frontline healthcare workers in low-income settings, with HIC partners taking structured responsibility for evidence synthesis and adaptation while LIC partners lead contextually grounded implementation and quality assurance. More broadly, sustainable partnerships require a shift from short-term project cycles to long-term commitment, from parallel service delivery to health system strengthening, and from external control to genuine local ownership. The ultimate measure of success is not by what international partners deliver, but by what continues, adapts, and spreads once they step back – a standard that this partnership’s national-trainer integration, cross-border transfer, and sustained registry use go some way toward meeting.

Strengths of this project include its grounding in a systematic, WHO-validated baseline assessment enabling targeted priority-setting; a multi-component design combining the COM-B framework and an embedded train-the-trainer model; and broad institutional engagement – the Ministry of Health, hospital leadership, NGOs, and regional politicians – supporting local ownership. Key limitations include insufficient prospective outcome data, limiting attribution of the observed case fatality reduction to partnership activities; staff turnover from mandatory rotation and migration, which diluted capacity gains; and a single-region focus limiting generalisability. Implementation outcomes (e.g. acceptability, adoption, appropriateness, feasibility, fidelity, penetration, sustainability) cannot be reported against a structured framework, as data were collected for programme monitoring rather than implementation research, without prospective instruments for these domains. Future evaluations of similar partnerships should incorporate structured implementation outcome measurement from the outset, guided by an established framework.

The Busoga–Stockholm partnership illustrates how twinning partnerships can create sustained, peer-to-peer relationships enabling knowledge transfer and co-adaptation of evidence to local contexts, with high potential to improve quality of care. Unlike short-term training, they build internal capacity through local trainers, institutional protocols, and empowered staff. Despite ongoing resource shortages, targeted training, improved routines, and enhanced staff motivation can make a substantial difference, and continued collaboration holds real potential for long-term improvements in children’s health.

## Data Availability

The data that support the findings of this study (training logs, results from the initial QoC assessment, etc.) are available from the corresponding author, [HH], upon reasonable request.
